# A short review on lymphatic endothelial cell heterogeneity

**DOI:** 10.1186/s41232-021-00183-6

**Published:** 2021-10-11

**Authors:** Masayuki Miyasaka

**Affiliations:** Immunology Frontier Research Center (IFReC), Osaka University, 3-3-1 Yamada-oka, Suita, Osaka, 565-0871 Japan

**Keywords:** Lymphatic endothelial cell, Heterogeneity, Microenvironment

## Abstract

Recent single-cell RNA sequencing studies in mouse and human have clearly indicated that lymphatic endothelial cells (LECs) consist of multiple cell subsets, each expressing a unique set of genes, residing in distinct locations in the body. These studies have also revealed a conserved pattern of gene expression in LECs across animal species, as well as specialized sets of genes unique to each species. However, the extent to which this heterogeneity is adaptive to the external milieu surrounding LECs has remained unclear. The transcriptional and regulatory pathways that program the different subsets of LECs also remain unexplored.

## Main text

Recent studies using single-cell transcriptomics of lymphatic endothelial cells (ECs) (LECs) have unambiguously shown that these cells are not just a homogeneous group of cells lining a conduit for fluid and cells, but actually consist of multiple cell subsets [1–4] that together serve a variety of physiological functions in different tissues. These functions include regulating lymphocyte/dendritic cell egress from {5] and ingress into lymph nodes [6, 7], antigen presentation [8], tolerance induction [9], and transport of lipoproteins and chylomicrons [10]. LECs are clearly active players in major physiological processes.

However, several issues concerning the previously described endothelial heterogeneity remain unclear. First, it remains unclear how these phenotypic differences between LEC subsets contribute to specific functions of lymphatic vessels. Second, we do not know how these differences are established and maintained. Given that endothelial cells are plastic and can adopt subtly different phenotypes in response to environmental stimuli, these phenotypic differences are likely to be governed at least in part by differences in the extracellular milieu. Because endothelial cells have the ability to sense and respond to their environment, the diversity of micro-anatomical signals between one region and the next may contribute to the generation of phenotypic heterogeneity within a tissue. As indicated by Lee et al. [11], plasticity is likely to be the main mechanism that enables heterogeneity. It also remains unclear to what extent these phenotypic differences are fixed. The relative contributions of epigenetic and non-epigenetic influences to the phenotypic heterogeneity need to be elucidated.

The results from recent studies on LEC heterogeneity using single-cell RNA sequencing (scRNA-seq) of lymph node-derived LECs in human and mouse are summarized in Table 1. A study by Takeda et al. [1] investigated the heterogeneity of human LN lymphatics by scRNA-seq of LECs isolated from surgically excised fresh axillary LNs, as well as head and neck LNs, identifying six types of LEC (LEC I–LEC VI). Interestingly, these LEC subtypes are not only transcriptionally different from each other but also located in different anatomical sites, as shown in Table 1. For instance, LEC1 is found in the ceiling of the subcapsular sinus and expresses *CCRL1*, which encodes the chemokine receptor CCRL1 (chemokine C-C motif receptor-like protein-1) or the atypical chemokine receptor ACKR4 (atypical chemokine receptor 4) that internalizes and thus scavenges the homeostatic chemokines CCL19, CCL21, and CCL25 in situ. Because these homeostatic chemokines are involved in regulating immune cell trafficking within LNs, the LEC I subset is likely to be involved in the regional regulation of immune cell trafficking. In contrast, LEC II is selectively found in the floor of the subcapsular sinus and expresses CCL20, which recruits innate-like lymphocytes to this region. LEC III is found in the LN capsule ceiling overlying the medulla. LEC IV is found in the head and neck LNs, but not in the axillary LNs. LEC V is found preferentially in the lymphatic valve, and LEC VI is selectively found in the medulla.

**Table 1:** LEC subsets in human and mouse LNs

Subset names			Localization	Uniquely expressed genes	Proposed functions
Human (Takeda et al.)	Mouse				
	#1 Xiang et al.	#2 Fujimoto et al.			
LEC I	cLEC	cLEC	Subcapsular sinus (ceiling)	*CCRL1, NT5E, CAV1* (human), *Ackr4, Foxc2* (mouse #1) *Ackr4, CD36, Ackr3, Bgn* (mouse #2)	Chemokine scavenging?
LEC II	fLEC	fLEC	Subcapsular sinus (floor)	*TNFRSF9, MARCO, CCL20* (human) *Madcam1, Ccl20, Cd274* (mouse #1) *Cd44, Glycam1, Coch* (mouse #2)	Chemokine Presentation/scavenging?
LEC III			Capsule ceiling overlying the medulla	*CCRL1, LYVE1, MFAP4* (human)	
LEC IV			Head and neck LNs	*CAV1, LYVE1* (human)	
LEC V	Valve		Lymphatic valve	*CAV1, NT5E, CLDN11* (human) *Ackr4, Foxc2*, *Ccl21a* (mouse #1)	
LEC VI	Marco-LEC	Medullary LEC	Medulla	*CLEC4G, CLEC4M, CD209* (human) *Cd274, Lyve1, Marco (mouse #1)* *Marco, Itga2b, Lyve1* (mouse #2)	Neutrophil trapping?
	Ptx3-LEC	Egress sinus LEC	Cortical/medullary sinuses	*Lyve1, CCL21a,Ptx3 (mouse #1)* *Ptx3, Kcnj8, Itih5* (mouse #2)	Lymphocyte egress?

*CCRL1* the human gene encoding the chemokine receptor CCRL1 (chemokine-C-C-motif receptor-like protein-1) or ACKR4 (atypical chemokine receptor 4), which binds dendritic cell- and T cell-activated chemokines including CCL19, CCL21, and CCL25

*NT5E* the human gene encoding the purine catabolic enzyme ecto 5′-nucleotidase (CD73), which is a plasma membrane protein that catalyzes the conversion of extracellular AMP (adenosine monophosphate) to adenosine

*CAV1* the human gene encoding a scaffolding protein, caveolin-1, which is the main component of caveola plasma membranes

*Ackr4* the mouse gene encoding the chemokine receptor ACKR4 (atypical chemokine receptor 4) or CC chemokine receptor type 11 (CCRL1)

*Foxc2* the mouse gene encoding a member of the Forkhead box (FOX) family of transcription factors, Foxc2. Mutation of this gene is observed in patients with lymphedema–distichiasis syndrome, which is characterized by late childhood- or pubertal-onset lymphedema of the limbs. Lymphatic valves and mural cell recruitment are thought to be defective in these patients

*CD36* the mouse gene encoding the scavenger receptor class B, member 3, which binds and internalizes oxidized low-density lipoprotein

*Ackr3* the mouse gene encoding the chemokine receptor AKCR3 (atypical chemokine receptor 3) or CXC chemokine receptor 7 (CXCR7), which can bind the chemokines CXCL12 and CXCL11

*Bgn* the mouse gene encoding biglycan, which is a small leucine-rich repeat proteoglycan (SLRP) found in the extracellular matrix of various tissues

*TNFRSF9* the human gene encoding TNF receptor superfamily member 9 (TNFRSF9), which is also called 4-1BB or CD137

*MARCO* the human gene encoding macrophage receptor with a collagenous structure (MARCO), which is a class A scavenger receptor that can bind modified low-density lipoproteins such as oxidized low-density lipoproteins

*CCL20* the human gene encoding CC motif chemokine ligand 20 (CCL20), which is strongly chemotactic for cells expressing the chemokine receptor CCR6

*Madcam1* the mouse gene encoding mucosal addressin cell adhesion molecule-1 (MAdCAM-1), which is an endothelial molecule that interacts with leukocyte alpha4beta7 (α4β7) integrin

*Cd274* the mouse gene encoding CD274 or programmed cell death 1 ligand 1 (PD-L1), which binds the receptor PD1 abundantly expressed on activated T cells and blocks T cell activation

*Cd44* the mouse gene encoding CD44, which is a cell-surface glycoprotein that plays a role in cell adhesion and migration

*Glycam1* the mouse gene encoding glycosylation-dependent cell adhesion molecule 1 (GlyCAM-1), a cell-surface protein that binds the leukocyte receptor L-selectin

*Coch* the mouse gene encoding cochlin, which is an extracellular matrix protein that binds collagen VII. Cochlin-deficient mice show a higher frequency of infection with *Pseudomonas aeruginosa* and *Staphylococcus aureus*

*LYVE1* the human gene encoding lymphatic vessel endothelial hyaluronan receptor 1 (LYVE1), which is a cell-surface glycoprotein that binds hyaluronic acid

*MFAP4* the human gene encoding microfibril-associated glycoprotein 4 (MFAP4), which is an extracellular matrix protein involved in intercellular interactions

*CLDN11* the human gene encoding claudin 11, which is a tight junction protein involved in the minimization of intercellular space

*Ccl21a* the mouse gene encoding CC motif chemokine 21 (CCL21), which induces chemotaxis of naïve T cells and dendritic cells by binding to the chemokine receptor CCR7

*CLEC4G* the human gene encoding C-type lectin domain family 4 member G (CLEC4G), which is a cell-surface C-type lectin receptor involved in innate immune responses

*CLEC4M* the human gene encoding C-type lectin domain family 4 member M (CLEC4M), which binds intercellular adhesion molecule-3 (ICAM-3) as well as the gp120 molecule of HIV-1

*CD209* the human gene encoding dendritic cell-specific intercellular adhesion molecule-3-grabbing non-integrin (DC-SIGN) or CD209, which binds to various pathogen-associated molecular patterns (PAMPs) found on viruses, bacteria, and fungi

*Itga2b* the mouse gene encoding integrin alpha chain 2b, which joins with integrin beta 3 chain to form a heterodimeric protein, α2β3 integrin

*Ptx3* the mouse gene encoding pentraxin-related protein 3 (PTX3), which is involved in the regulation of innate resistance to pathogens

*Kcnj8* the mouse gene encoding potassium inwardly rectifying channel, subfamily J, member 8 (KCNJ8)

*Itih5* the mouse gene encoding inter-alpha-trypsin inhibitor heavy chain 5 (ITIH5)

Studies using mice have also identified five to six different LEC subsets, as summarized in Table 1. Apart from those identified in humans [1], one study [2] found an LEC subset that covers the inner surface of the cortical to medullary sinuses, preferentially expressing *Lyve1*, *CCL21a*, and *Ptx3*. Judging from its gene expression profile and anatomical location, this subset appears to be identical to the LEC subset implicated in rapid lymphocyte egress from LNs [3].

Clearly, further study is required to functionally delineate these LEC subsets. In addition, their ontogeny, the lineages among these subsets, and the molecular requirements for the development of these subsets also remain to be studied.

## Conclusions

In summary, while a number of LEC subsets have been defined in lymph nodes on the basis of their gene expression patterns and anatomical locations, further investigation is required to fully understand their physiological and pathological roles in lymphoid tissues. It would also be interesting to examine how infections, cancer, and other diseases affect LEC heterogeneity in lymphoid tissues.

## Data Availability

Not applicable

## Abbreviations

LEC: Lymphatic endothelial cell
